# Proof of principle study replicating microbial clusters in connection to birth mode and diet in the early life intestine

**DOI:** 10.1371/journal.pone.0277502

**Published:** 2022-11-11

**Authors:** Patrick Schimmel, Lennart Kleinjans, Carl Vael, Kristine Desager, Jan Knol, Clara Belzer

**Affiliations:** 1 Laboratory of Microbiology, Wageningen University & Research, Wageningen, The Netherlands; 2 Laboratory of Microbiology, Klina Hospital, Brasschaat, Belgium; 3 Private Clinic, Aartselaar, Belgium; 4 Danone Nutricia Research, Utrecht, The Netherlands; University of Minnesota Twin Cities, UNITED STATES

## Abstract

The human gut ecosystem starts developing at birth and is influenced by many factors during early life. In this study we make use of a Belgian cohort of 64 children, followed until the age of 6 years, to analyze different phases of microbiota development. We analyzed fecal samples taken before weaning (age 1 month), shortly after weaning (age 6 months), when milk feeding has been discontinued completely (age 1 year), and at the age of 6 years. We performed 16S rRNA gene amplicon sequencing on the collected fecal samples and analyzed the compositional data in relation to dietary metadata and birth mode. Human and formula milk feeding promotes a microbiota dominated by either *Bacteroides* or *Bifidobacterium*, respectively. Into later life stages, the microbiota composition follows distinct microbiota clusters, related to abundance dynamics of certain bacterial groups. Furthermore, it becomes apparent that a formula diet leads to early maturation of the infant gut microbiota. Despite other clinical variables within the infant cohort, they did not significantly contribute to the microbiota patterns we observed. Our data provide a proof of principle study of the importance of diet to the development of the microbiota in early life that replicates earlier findings in other cohorts.

## Introduction

The seeding and development of the human gut microbiota has been implicated to be involved in health outcomes during early and later life. Examples of diseases related to a disrupted microbiota homeostasis are asthma, allergy, eczema, obesity and inflammatory bowel disease [1–3]. Over recent years, there have been many reports on the microbiota of infants with the use of multiple methods and samples taken at a multitude of ages [4–11]. However, limited data is available on longitudinal cohort studies of the gut microbiota of children. The development of the gut ecosystem intensifies at birth when a child encounters the mothers’ vaginal and faecal microbiota as it exits the birth canal, which can be affected for infants born through ceasarian section (C-section) [12, 13]. However, recent studies have shown when studying and correcting for maternal comorbidities in relation to the occurrence of a birth mode effect on the infant gut microbiota, that such an effect does not exist [14]. Postnatally, facultative anaerobic bacteria from the genera *Streptococcus* [15], *Enterobacteriaceae* [16], *Bacillus* [17], are commonly detected in the infant gut and colostrum [18]. Once an anaerobic environment is ensured, the microbiota becomes more stable with genera such as *Bacteroides* spp. and *Bifidobacterium* spp. occurring in higher relative abundance which are maintained throughout milk feeding [15–17, 19]. Human milk and infant formula both contain oligosaccharides indigestible to humans. Gut bacteria specialized in utilizing these oligosaccharides as growth substrate therefore often become dominant in the infant gut microbiota. The most well established degraders of human milk oligosaccharides (HMOs) are members of the genus *Bifidobacterium*. Some bifidobacteria are highly specialized in metabolizing HMOs [20] and *Bifidobacterium* spp. are therefore often dominant in the infant microbiota [2, 15]. However, also *Bacteroides* spp. are known to degrade HMOs [21]. A few years ago, a *Bacteroides-*dominated infant microbiota was discovered [13, 19, 22]. The *Bacteroides-*dominated microbiota has been described in relation to birth mode. Vaginally born infants have been reported to have higher levels of *Bacteroides* spp. and meanwhile C-section born infants have been described to be depleted of *Bacteroides* spp., although both cannot be exclusively linked to birth mode [16, 19]. Vatanen *et al*. describe that the microbiota of children from Estonia and Finland is dominated by *Bacteroides* during infancy. This contrasts with the microbiota of Russian infants, which is first initially occupied by *Bifidobacterium* spp. and only later transitions to *Bacteroides* spp. becoming more prevalent [23]. Typically, breastfed infants have lower microbial diversity than formula fed infants [16]. The breastfed infant’s microbiota is often enriched in more typical glycan degraders such as *Bifidobacterium* spp., whereas the formula fed microbiota was enriched in *Clostridia* spp. and *Enterobacteriaceae* spp. [15, 16]. Because human milk has such a profound effect on microbiota composition, a major shift in microbiota composition occurs when milk feeding is discontinued. Typical adult-associated bacteria like *Roseburia* spp., *Bilophila* spp., *Anaerostipes* spp. and *Clostridium* spp. increase significantly in abundance [15]. This indicates clusters of microbiota composition occurring in early life between infants with different lifestyles. This fragmented and diverse development of the early life gut microbiota is also studied here in a longitudinal cohort. Some of the most important biomarkers for the aging gut microbiota were *Faecalibacterium prausnitzii* and a species of *Ruminococcus*, since they were positively correlated with age [24]. In terms of diversity parameters, it seems that the gut microbiota reaches an adult-like composition at the age of about 3–4 years [5].

We have access to a comprehensive sample collection of faecal samples from a Cohort of Belgian children, taken at crucial time-points (1 month, 1 year, 6 years) from groups of children with different diets. This provides a longitudinal cohort with some typical aspects of human gut microbiota development in early life. We investigated the effects of birth mode, feeding mode and duration on microbiota composition, with a focus on developmental dynamics and maturation. 16S rRNA amplicon sequencing was performed on collected fecal material and a bio-informatics analysis on the compositional data and metadata was conducted.

## Methods

### Clinical trial setup and samples

**Fig 1:**
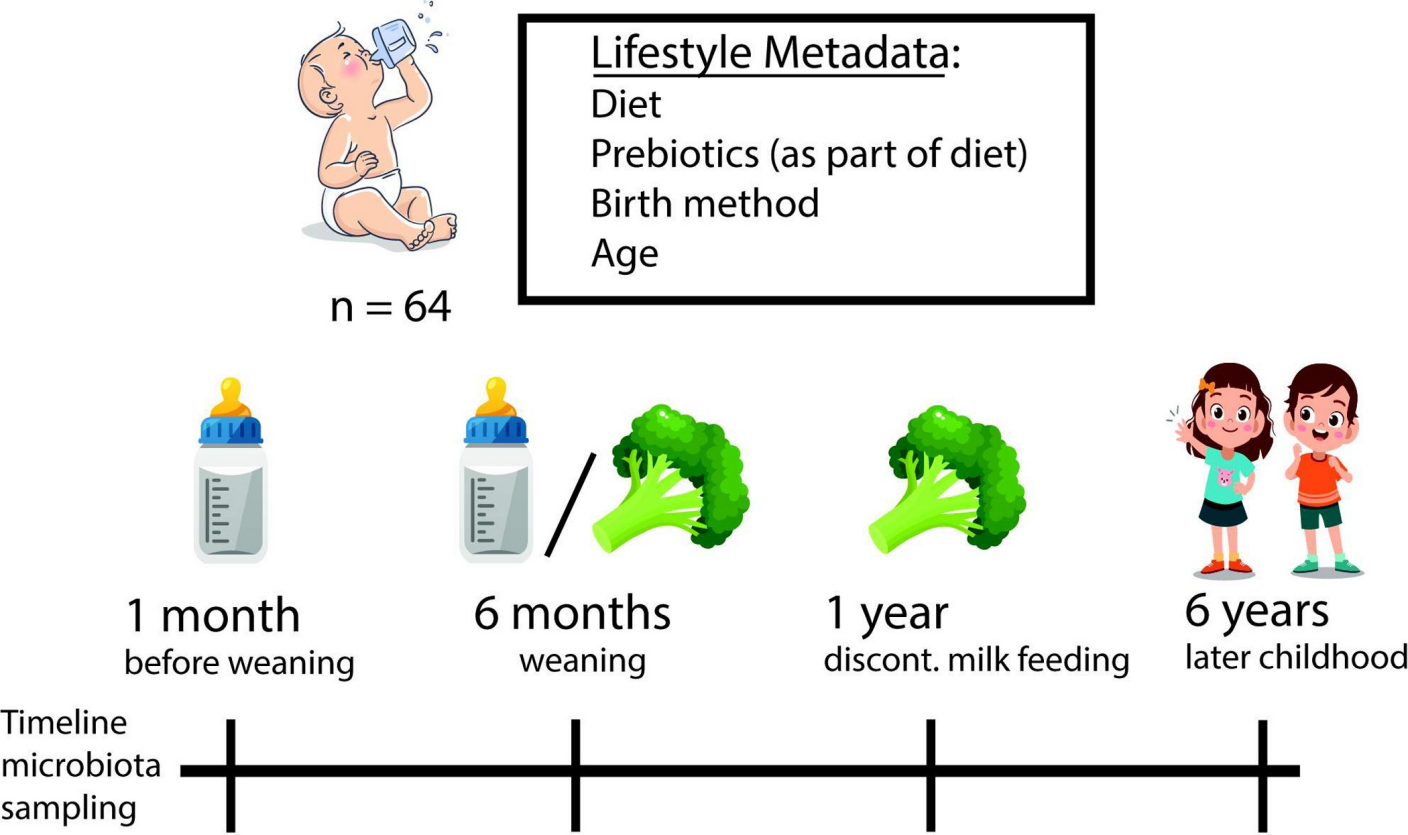
NTR3505 study setup. Study setup & metadata (birth mode, feeding, age, prebiotic supplementation).

The faecal samples analysed in this study originate from a observational trial (follow-up study registered as NTR3505, 2012), that was ethically approved at the Commissie Medische Ethiek (medical ethical committee, July 12^th^, 2010) in Antwerp, Belgium. The study was not registered in the trial register at the time as the study started before the trial registration was standard procedure. The aim was to investigate whether intervention with prebiotics during the first year of life has an impact on prevalence of atopic diseases at 6 years of age. Both parents gave written consent for inclusion. The study included 64 children (inclusion 2004–2006)., at high risk for atopy (parental history), followed until the age of 6 years. It was performed in a randomized; controlled, double-blind parallel-group way. It was tested if the incidence of allergic disease can be reduced through the use of hydrolyzed infant formula with short-chain galacto-oligosaccharides and long-chain fructo-oligosaccharides (GOS/FOS) together with pectin-derived acidic oligosaccharides (pAOS). Infants either received a hypo-allergenic formula with or without a mixture of GOS/FOS and pAOS until 1 year of age. Mothers were encouraged to breastfeed as long as possible. Describing the infant gut microbiota was a part of the study design, however the specific bioinformatics methodology was considered post-hoc. Our data describes several lifestyle variables in relation to the gut microbiome and its development in this European infant cohort (Fig 1).

### Bacterial 16S rRNA gene amplicon sequencing

Samples were prepared for Illumina Miseq sequencing using a two-step protocol to amplify the 16S rRNA and to barcode the samples. Bacterial 16S rRNA gene fragments were amplified by using universal primers covering the V3-V4 region of the bacterial 16S rRNA gene. The forward primer consisted of the S-D-Bact-0341-b-S-17 primer (5’-CCTACGGGNGGCWGCAG-3’) [25] added to the 3’ end of the Unitag1 barcoding adapter (5’-GAGCCGTAGCCAGTCTGC-3’). The reverse primer consisted of the S-D-Bact-0785-a-A-21 primer (5′-GACTACHVGGGTATCTAATCC-3’) [25] added to the 3’ end of the Unitag2 barcoding adapter (5’-GCCGTGACCGTGACATCG-3’). The PCR was performed in a volume of 50μL containing 10μl of 5× HF green buffer (Finnzymes, Vantaa, Finland), 1μL dNTP mix (Promega Benelux B.V., Leiden, The Netherlands), 0.5μL of Phusion Hot Start II DNA polymerase (2U/μl; Finnzymes), 2.5μL of the reverse primer mix and the forward primer (both 10μM), 1μL template, and 32.5μL nuclease free water. The PCR program was 98°C for 30 seconds to activate the enzyme, then 25 cycles of 98°C for 10 seconds, 56°C for 20 seconds, 72°C for 20 seconds, and then a final extension at 72°C for 10 minutes.

The PCR products were analysed on a 1.2% FlashGel (Lonza, Basel, Switzerland) to verify product formation. If a clear band was visible, 5μL of PCR product was used in a second PCR with 8 nucleotide sample specific barcodes, which were added to the Unitag1 and Unitag2 sequences. This second PCR mixture contained 20μL HF green buffer (Finnzymes), 2μL dNTP mix (Promega), 1μL Phusion Hot Start II DNA polymerase (Finnzymes), 62μL nuclease free water, 5μL forward barcoded Unitag1 primer (10μM), 5μL reverse barcoded Unitag2 primer (10μM) and 5μL product of the first PCR. The PCR program started with an activation step at 98°C for 30 seconds, followed by 5 cycles of 98°C for 10 seconds, 52°C for 20 seconds, 72°C for 20 seconds, and finished with an extension step at 72°C for 10 minutes. Product formation was verified on a 2.2% Flashgel (Lonza). The PCR product was purified using the Highprep PCR clean-up magnetic beads (Magbio, London, UK). The concentration of the cleaned PCR product was measured with the Qubit dsDNA BR Assay Kit in the Qubit 2.0 device (Thermo Fischer, Waltham, MA, USA). Finally, the samples were pooled equimolarly with 48 samples per library, including 2 mock communities as an internal standard. Then the libraries were concentrated with the Highprep PCR beads (Magbio). The samples were analysed on the Illumina HiSeq sequencing platform in Rapid Run mode. (Illumina, San Diego, CA, USA).

### Data analysis

The raw sequencing data were analysed with the in-house NG-tax pipeline and using its final taxonomic labels [26]. Briefly, paired-end libraries were filtered to contain only read pairs with perfectly matching barcodes. These barcodes were used for demultiplexing. Operational Taxonomic Unit (OTU) picking was performed with an open reference approach and a customized SILVA 16S rRNA gene reference database [27]. ClustalW was used to generate an alignment of OTU sequences and corresponding dendogram. Samples with less than 1000 reads were filtered from the OTU table. Alpha and beta diversity metrics were calculated using scripts from the Quantitative Insights Into Microbial Ecology (QIIME) v1.8.0 package [28]. Ordination analyses were performed with the Canoco 5.0 software package [29]. Age types were determined by Partitioning Around Medoids (PAM) clustering, based on a weighted UniFrac [30] distance matrix. The clustering analysis was performed in R version 3.2.3 and the total number of infants available at each timepoint was modelled into the trajectories (n = 49). Statistical analyses were performed in SPSS version 22.

## Results

### Ordination analyses reveal taxa that characterise microbiota aging

**Fig 2:**
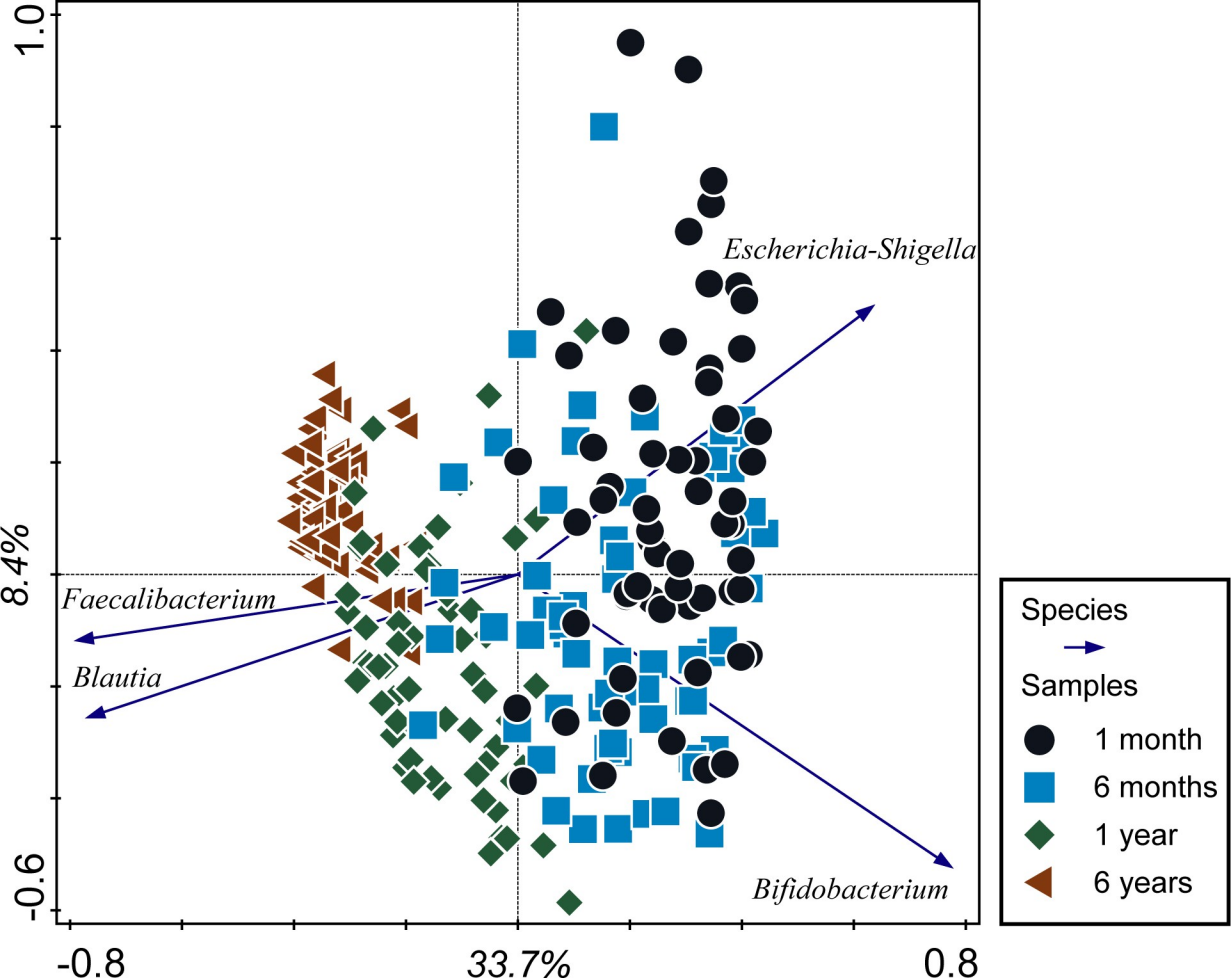
Principal component analysis. Principal component analysis based on microbiota composition for all samples, labelled per timepoint.

First we performed a Principal Component Analysis (PCA) based on the microbiota composition of all samples from all time points (Fig 2). The analysis shows that a large proportion of the variance in microbiota composition can be explained by age. This is illustrated by the 1-month and 6-year samples positioned on the extremes of the first principal component. There is a large overlap between 1m and 6m samples, yet a fraction of the 1m samples do not overlap with 6m samples. These samples are characterized by a high abundance of *Escherichia-Shigella* spp., whereas the 1m samples that overlap with 6m samples are characterized by *Bifidobacterium* spp. The later time points are characterized by increasing numbers of *Faecalibacterium* spp. and *Blautia* spp.

**Fig 3:**
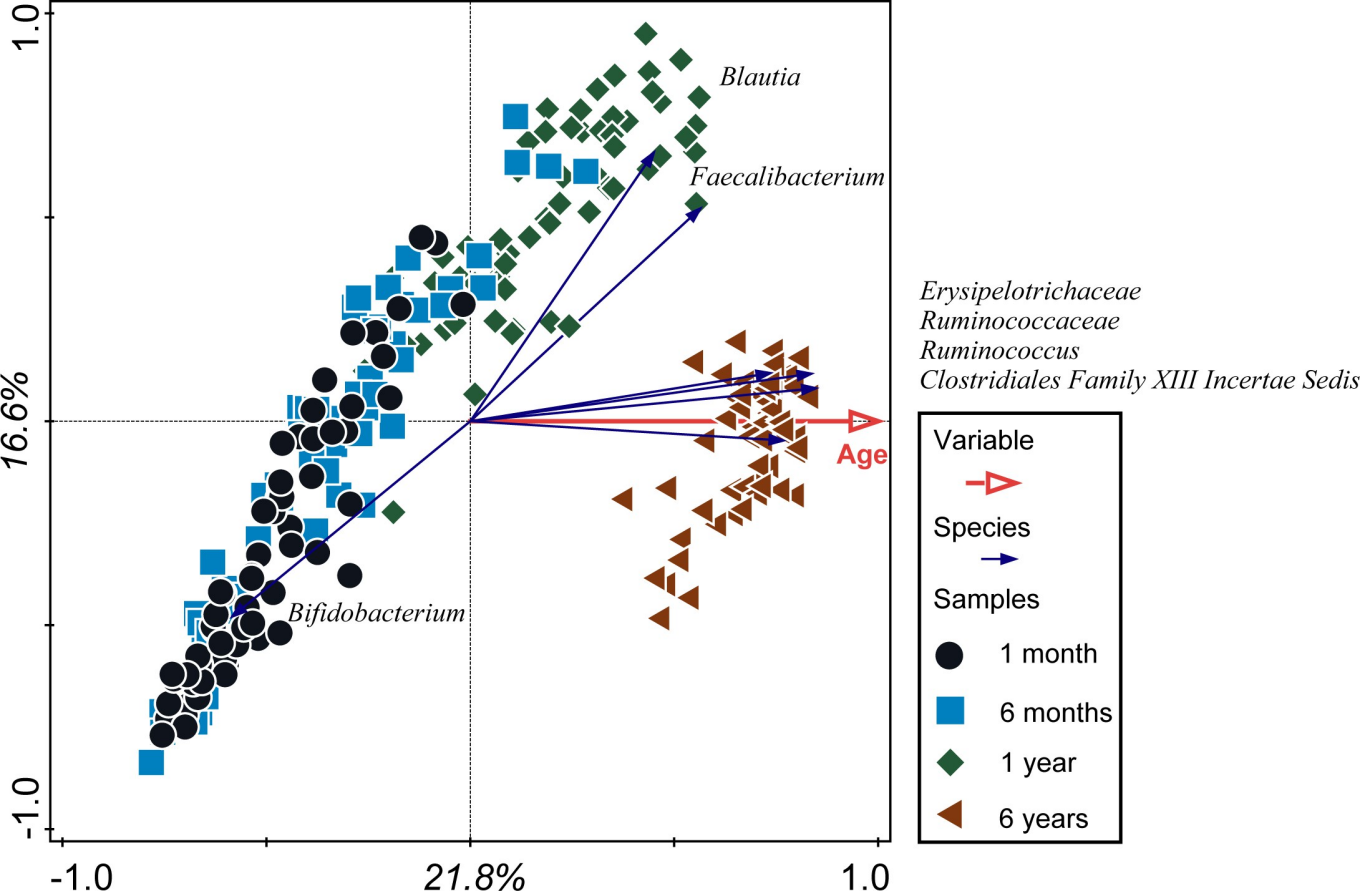
Redundancy analysis (RDA). RDA on all included samples based on microbiota composition, with the main variable: age on the X-axis.

That the factor age is driving microbiota composition is further confirmed by a Redundancy Analysis (RDA). Age explains 21.8% of the observed variation in microbiota composition (Fig 3). It becomes apparent that increasing numbers of *Blautia* spp. and *Faecalibacterium* spp. are associated with increasing age between 1m and 1y. *Erysipelotrichaceae* spp, *Ruminoccaceae spp*, *Ruminococcus* spp and *Clostridiales Family XIII Incertae Sedis* are typical for the 6y microbiota. We compared the Spearman correlations of these taxa with age to their position on the X-axis of the PCA and RDA (Table 1). The Spearman correlations show an age effect, which is influenced strongly by the increase towards age 6y. The taxa X-axis values of the RDA also reflect the increase in abundance towards 6y, because age is used as an explanatory value in the analysis. The PCA X-axis values rather reflect microbiota maturity independently from the value of age. As such, taxa that have a higher PCA X-axis value as compared to the Spearman correlation, such as *Blautia* spp., *Dorea* spp. and *Faecalibacterium* spp. are typical for 1y samples. Similarly, the taxa that show higher RDA X-axis values than Spearman correlations are typical for the 6y samples, e.g. *Christensenellaceae* spp., *Clostridiales Family XIII Incertae sedis* spp., *Alistipes* spp., *Erysipelotrichaceae* spp., *Ruminococcus* spp. and *Ruminococcaceae* spp.

**Table 1:** A comparison of spearman correlations.

Taxon	RDA	PCA	Age correlation
*Bifidobacterium*	-0,584	0,773	-0,586
*Escherichia-Shigella*	-0,46	0,633	-0,595
*Veillonella*	-0,459	0,528	-0,529
*Streptococcus*	-0,417	0,566	0,628
*Staphylococcus*	-0,307	0,483	-0,666
*Anaerostipes*	0,399	-0,7	0,723
*Blautia*	0,45	-0,767	0,641
*Peptostreptococcaceae Incertae Sedis*	0,457	-0,427	0,514
*Coprococcus*	0,534	-0,512	0,608
*Lachnospiraceae*	0,535	-0,695	0,708
*Faecalibacterium*	0,564	-0,787	0,756
*Turicibacter*	0,616	-0,437	0,514
*Pseudobutyrivibrio*	0,628	-0,631	0,666
*Roseburia*	0,631	-0,689	0,717
*Dorea*	0,661	-0,684	0,662
*Subdoligranulum*	0,682	-0,656	0,682
*Alistipes*	0,694	-0,62	0,675
*Ruminococcaceae Incertae Sedis*	0,712	-0,66	0,726
*Christensenellaceae*	0,72	-0,484	0,657
*Erysipelotrichaceae*	0,736	-0,653	0,715
*Clostridiales Family XIII IncertaeSedis*	0,768	-0,577	0,663
*Ruminococcaceae*	0,836	-0,75	0,827
*Ruminococcus*	0,848	-0,735	0,806

A comparison of Spearman correlations with age and their positions on the X-axis of the PCA and RDA.

### Microbiota diversity does not increase during weaning

The availability of samples taken from the same infant at different time points allowed us to study the differences in alpha diversity over time. When alpha diversity is assessed as OTU richness, there is no significant difference between 1m (32.9) and 6m (35.3) samples (paired t-test, p = 0.163, Table 2). The difference in Simpson’s diversity between 1m (25.2) and 6m (27.7) samples is also not significant (paired t-test, p = 0.115). However, when alpha diversity is measured as phylogenetic diversity, the difference between 1m (1.345) and 6m (1.564) is significant (paired t-test, p = 0.001). This indicates that while the microbiota of the individuals is similar in richness and evenness, the microbial composition has changed. From 6m to 1y there was a large increase in richness (35.3 vs. 42.1, paired t-test, p<0.001). Simpson’s diversity increased from 27.7 to 41.6 (paired t-test, p<0.001), as well as phylogenetic diversity (1.551 vs. 2.027, paired t-test, p<0.001).An even larger difference in alpha diversity was observed between the samples taken at 1y and 6y. Richness increased from 52.1 to. 88.6 (paired t-test, p<0.001), Simpson’s diversity from 41.6 to 71.9 (paired t-test, p<0.001) and phylogenetic diversity from 2.037 to 3.241 (paired t-test, p<0.001).

**Table 2:** Diversity over time.

						Difference with previous age (95% C.I)			
Diversity index	Age	Mean	Min.	Max.	Std. Dev.	Mean	Lower	Upper	p-value
Simpson	1m	25,2	13,8	44,4	7	-	-	-	-
	6m	27,7	12,7	52,7	8,4	2,1	-0,5	4,8	0,115
	1y	41,6	24	65,1	9,1	14,2	11,4	17,1	<0,001
	6y	71,9	53,3	96,4	11	30,1	26,5	33,6	<0,001
OTU richness	1m	32,9	17	58	8,8	-	-	-	-
	6m	35,3	16	64	9,9	2,4	-1	5,7	0,163
	1y	52,1	29	80	10,9	17,2	13,8	20,5	<0,001
	6y	88,6	67	118	12,9	36,1	31,9	40,3	<0,001
Phylogenetic diversity	1m	1,35	0,68	2,28	0,36	-	-	-	-
	6m	1,56	0,86	2,58	0,38	0,21	0,09	0,34	0,001
	1y	2,04	1,34	2,66	0,32	0,48	0,36	0,59	<0,001
	6y	3,26	2,43	4,67	0,52	1,2	0,107	1,34	<0,001

Diversity metrics across all the timepoints for different models and the differences with the previous study timepoint.

### There are distinct early life microbiota clusters

**Fig 4:**
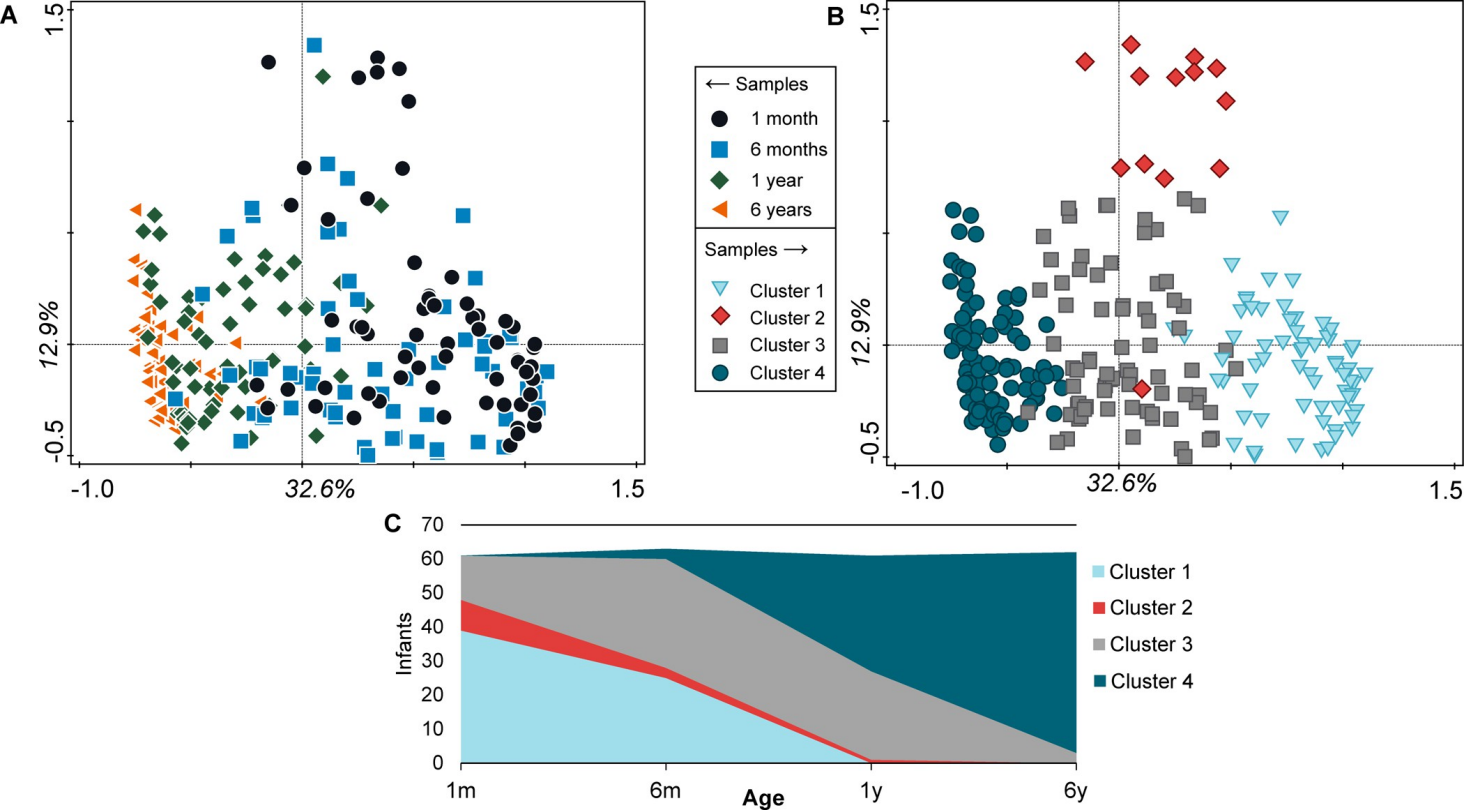
**(a) Principal Coordinates Analysis (PCoA).** PCoA based on Weighted Unifrac; (b) PAM-clustering with 4 clusters; (c) area-plot showing age-distribution within the clusters.

The age gradient observed in the PCAs also becomes apparent in a Principal Coordinates Analysis (PCoA) based on weighted UniFrac distances (Fig 4A). The samples taken at the 1m and 6y time points are on the outer ends of the first axis. The neighbouring time points show overlap, yet to a lesser extent between the 1y and 6y samples. To determine this overlap and further elaborate on stages in microbiota development we performed PAM-clustering based on weighted UniFrac distances. This resulted in four unique clusters (Fig 4B). An area plot shows the distribution of age over the different clusters (Fig 4C). There are two early life clusters, cluster 1 and 2. Cluster 1 consists of mainly 1m samples combined with about half of the 6m samples. Cluster 2 contains a lower number of samples, but also consists of mainly 1m and a few 6m samples. Cluster 3 can be considered an intermediate cluster, consisting of 6m and 1y samples with a few 1m and 6y samples. Cluster 4 contains almost all of the 6y samples, combined with about half of the 1y samples; therefore we named it the matured cluster.

### The early life microbiota is mostly dominated by either *Bacteroides* spp. or *Bifidobacterium* spp.

**Fig 5:**
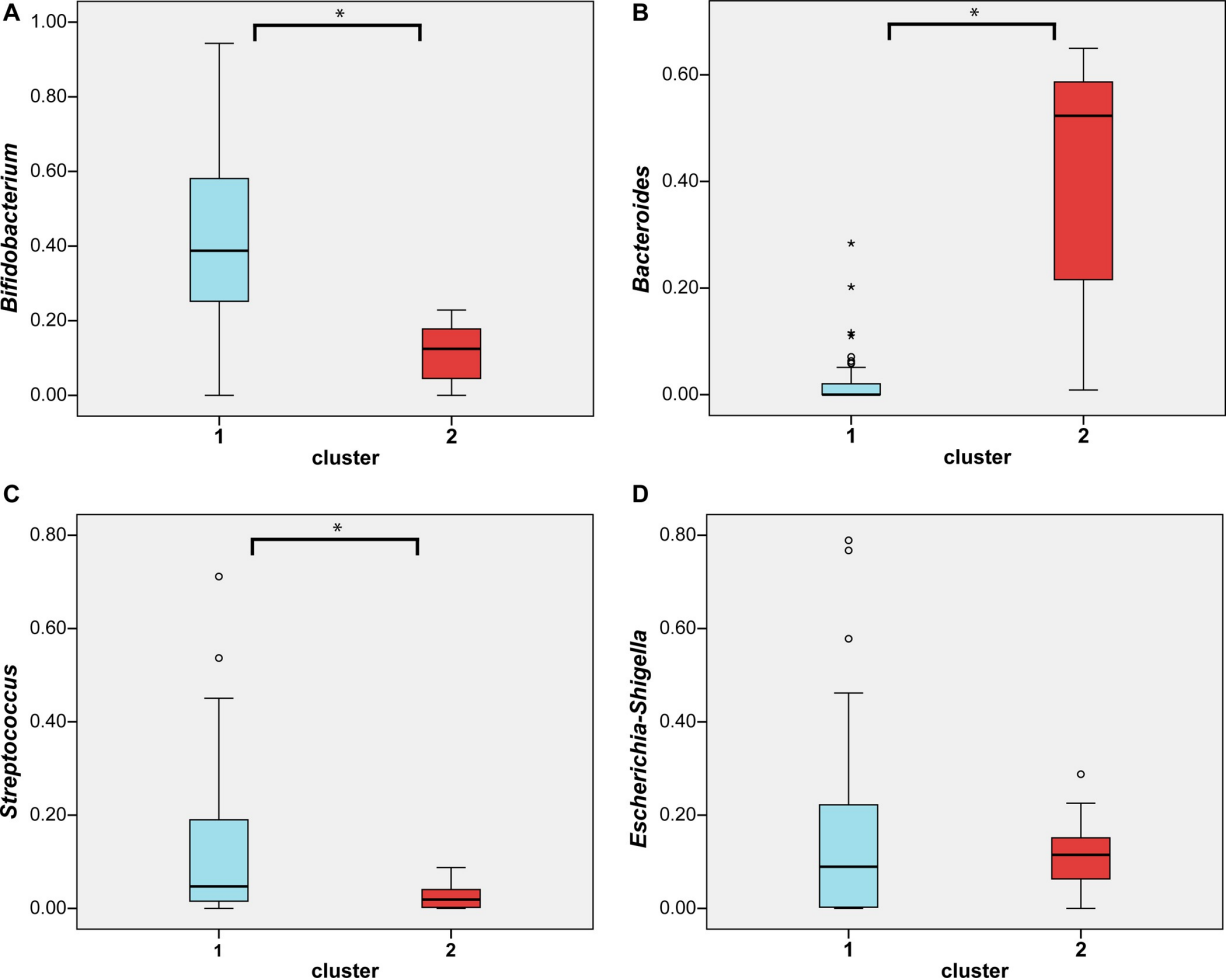
Relative abundance boxplots. Boxplots depicting bacterial difference (relative abundance) between the clusters.

The most common early life cluster, cluster 1, is dominated by *Bifidobacterium*. On average *Bifidobacterium* makes up 41.5% of the microbiota in cluster 1 (Fig 5). Cluster 2 however, is not dominated by *Bifidobacterium*; it only has an average relative abundance in this cluster of 11.0%. Instead, the dominant genus in cluster 2 is *Bacteroides*, with an average abundance of 40.0%. In cluster 1 *Bacteroides* is not at all common because it only makes up 2.2% of the total microbiota. The second most common genus in cluster 1 is *Streptococcus*, with an average abundance of 12.3%. That makes it significantly more abundant in cluster 1 than in cluster 2, which has an average of 2.6%. Also common in the early life microbiota are the facultative anaerobic *Proteobacteria*. In our study, the most abundant group in both clusters is *Escherichia*-*Shigella*, with comparable abundances of 14.4% for cluster 1 and 11.3% for cluster 2. The phylogenetic diversity of both early life clusters is very similar with values of 1.364±0.372 for cluster 1 and 1.367±0.194 for cluster 2.

### No *Bacteroides*-dominated microbiota in infants delivered through caesarean section

**Fig 6:**
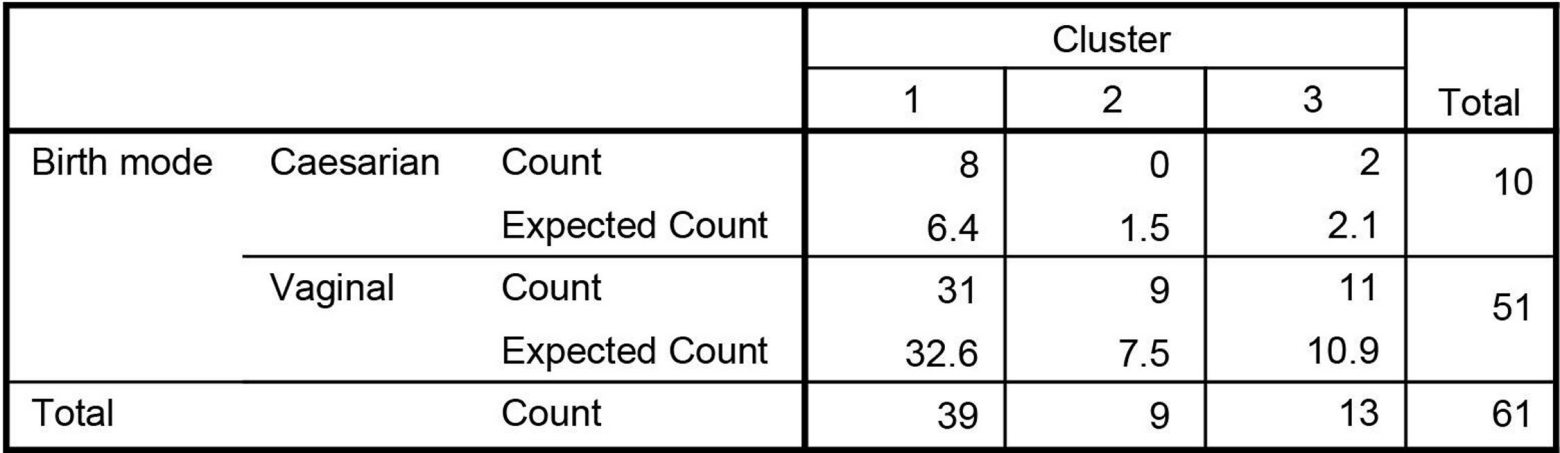
Cluster metadata. Birth mode metadata occurrence across the clusters.

The samples taken at 1m can be found in cluster 1, 2 and 3. Because delivery mode has previously been correlated with infant microbiota composition, we investigated whether the distribution of delivery mode was similar over the different clusters (Fig 6). At 1m 63.9% of the infants can be found in cluster 1, 14.8% in cluster 2 and 21.3% in cluster 3. The distribution of the clusters is very similar in infants born through vaginal birth, but differs in infants born through caesarean section. This different distribution is caused by the absence of infants from cluster 2 amongst the C-section born children. The number of infants however makes it impossible to perform a valid statistical test.

### There are four repeated developmental trajectories

**Fig 7:**
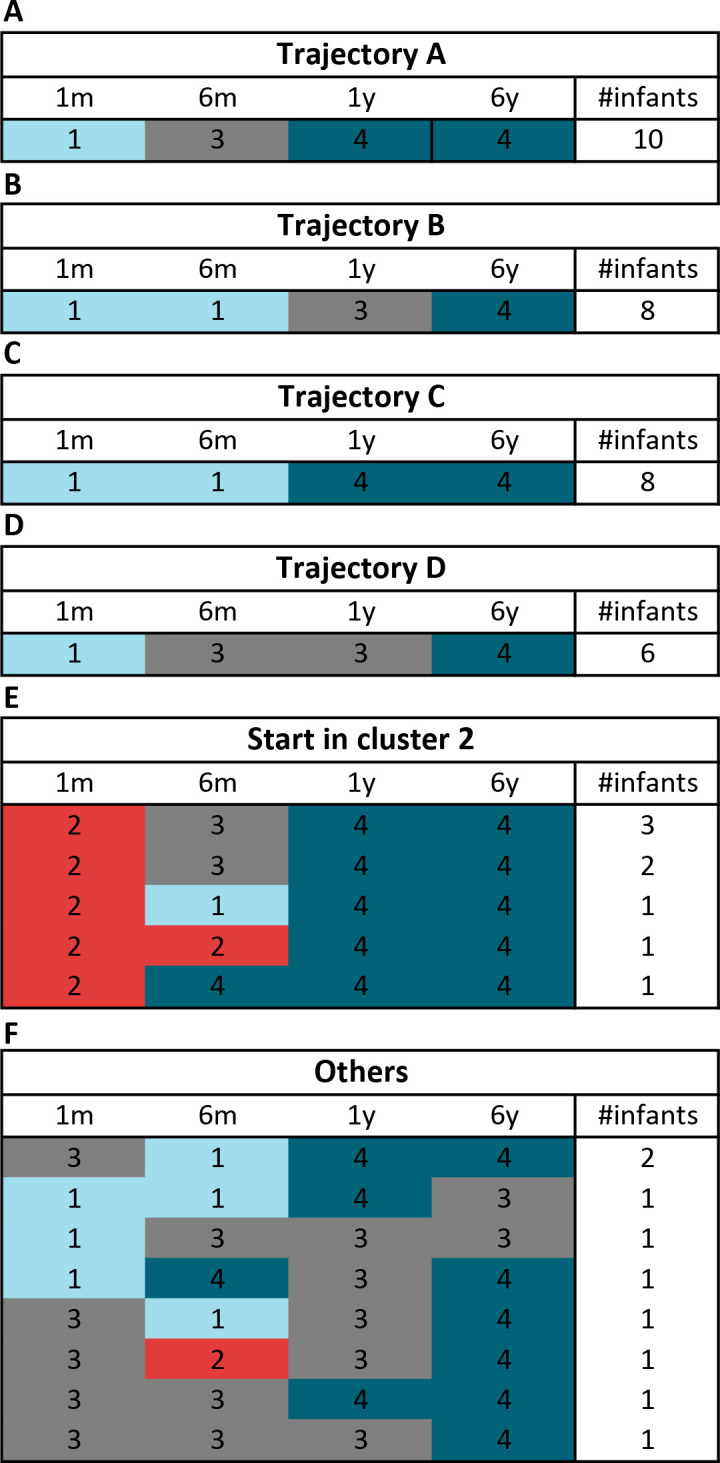
Trajectory formulations. Formulations and their occurrence rate across the infants.

**Fig 8:**
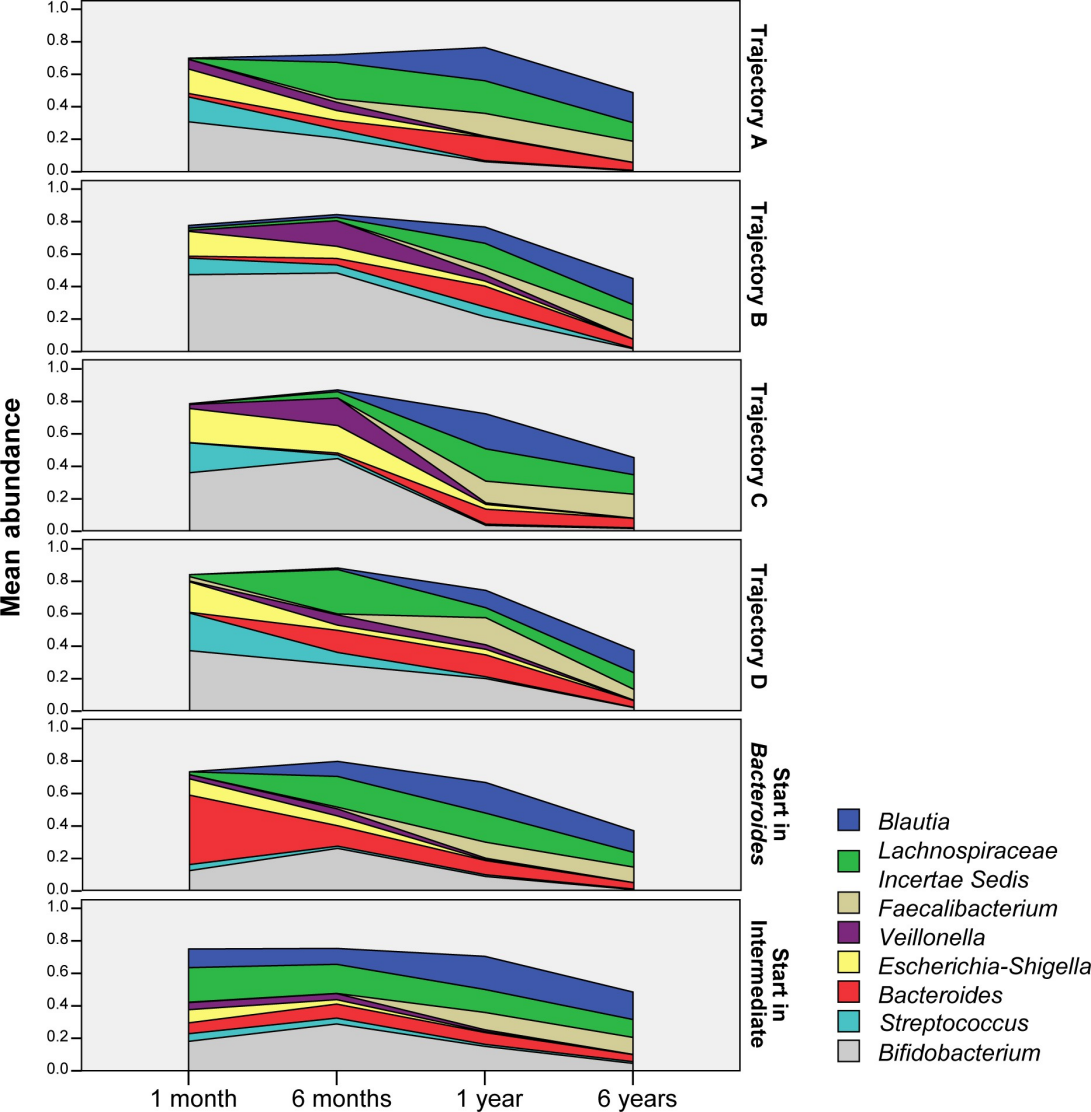
Trajectory area charts. Area charts showing the development of the relative abundances of the major bacterial groups across the major trajectories observed in this study.

The clustering analysis allows us to study microbiota maturation by following how individuals move through the clusters as age progresses. The most commonly observed progression is repeated 10 times (trajectory A). In trajectory A, infants are in cluster 1 at 1m, in cluster 3 at 6m and reach the matured cluster 4 at age 1y (Fig 7A). Trajectory B and C are the second most often repeated with 8 individuals each (Fig 7B and 7C). In both trajectories infants are in cluster 1 at both 1m and 6m. In trajectory B infants go through intermediate cluster 3 at 1y before reaching cluster 4 at 6y. In contrast to trajectory C, where the intermediate cluster is skipped and infants reach cluster 4 at the age of 1y. Trajectory D is observed in six individuals that are found in cluster 1 at 1m, cluster 3 at 6m and 1y before arriving in cluster 4 at 6y (Fig 7D). For individuals found in cluster 2 at 1m, there are only two repeated sequences (Fig 7E). With three individuals, the most repeated trajectory starting with cluster 2 is similar to trajectory A except for start. Notably, In trajectory B and C there is an increase in *Veillonella* spp. from 1m to 6m, whereas in A and D there is an increasing abundance in *Lachnospiraceae Incertae Sedis* spp. (Fig 8). The levels of *Bacteroides* in infants that are in cluster 2 at 1m restore to levels seen in other trajectories.

### Formula fed infants are more likely to have early microbiota maturation

**Fig 9:**
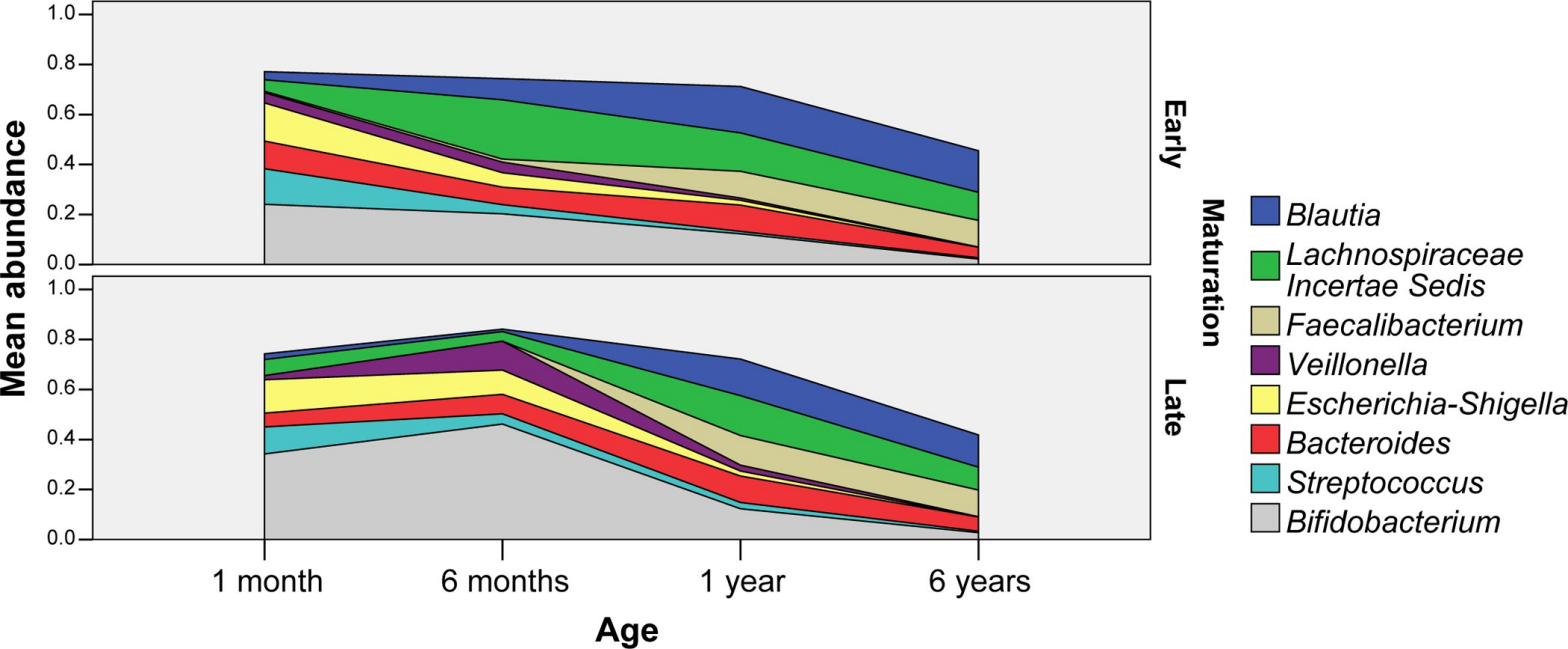
Abundance area charts. Area charts depicting relative abundance of major bacterial groups when separating infants into ‘early’ and ‘late’ maturation.

**Fig 10:**
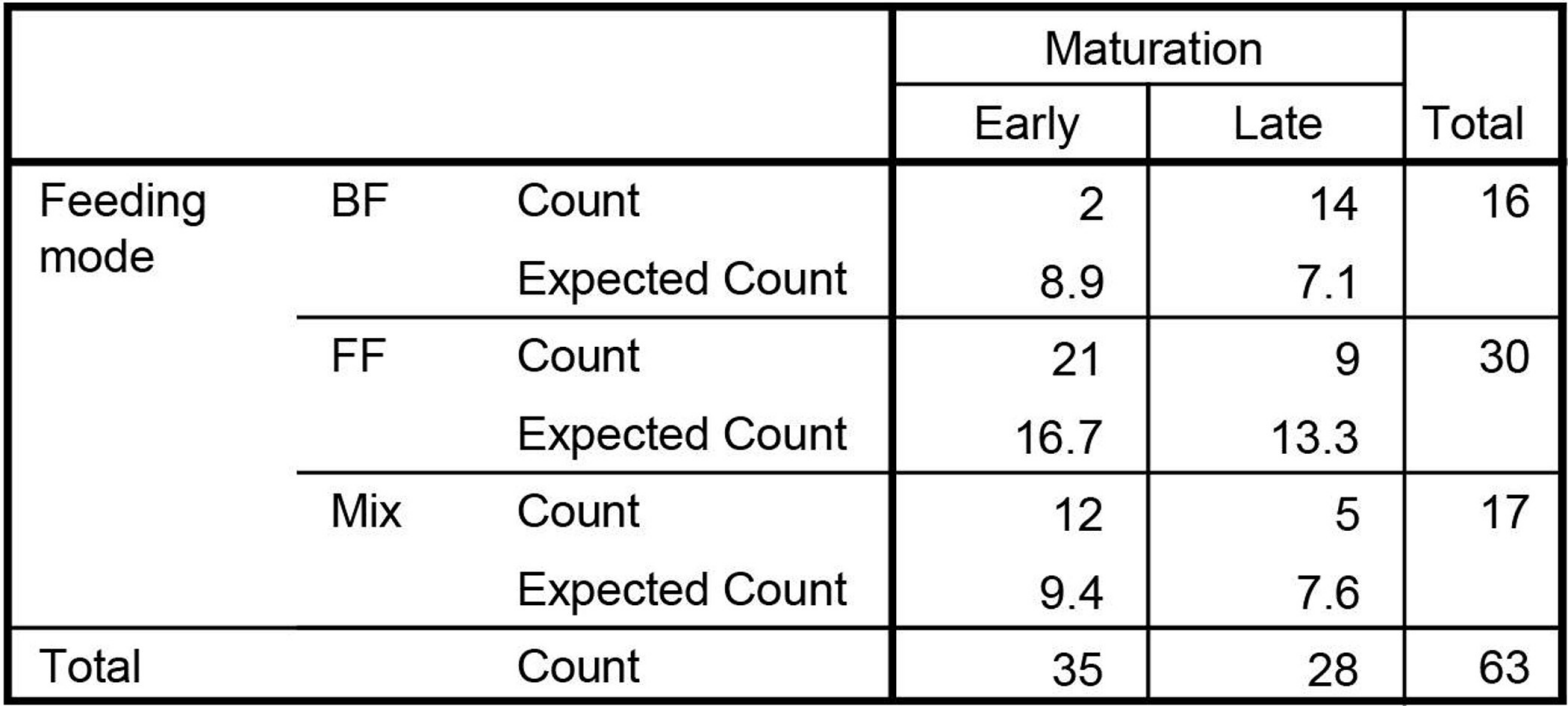
Maturation rates. The differences in maturation rate between feeding mode and the expected values if it was random.

**Fig 11:**
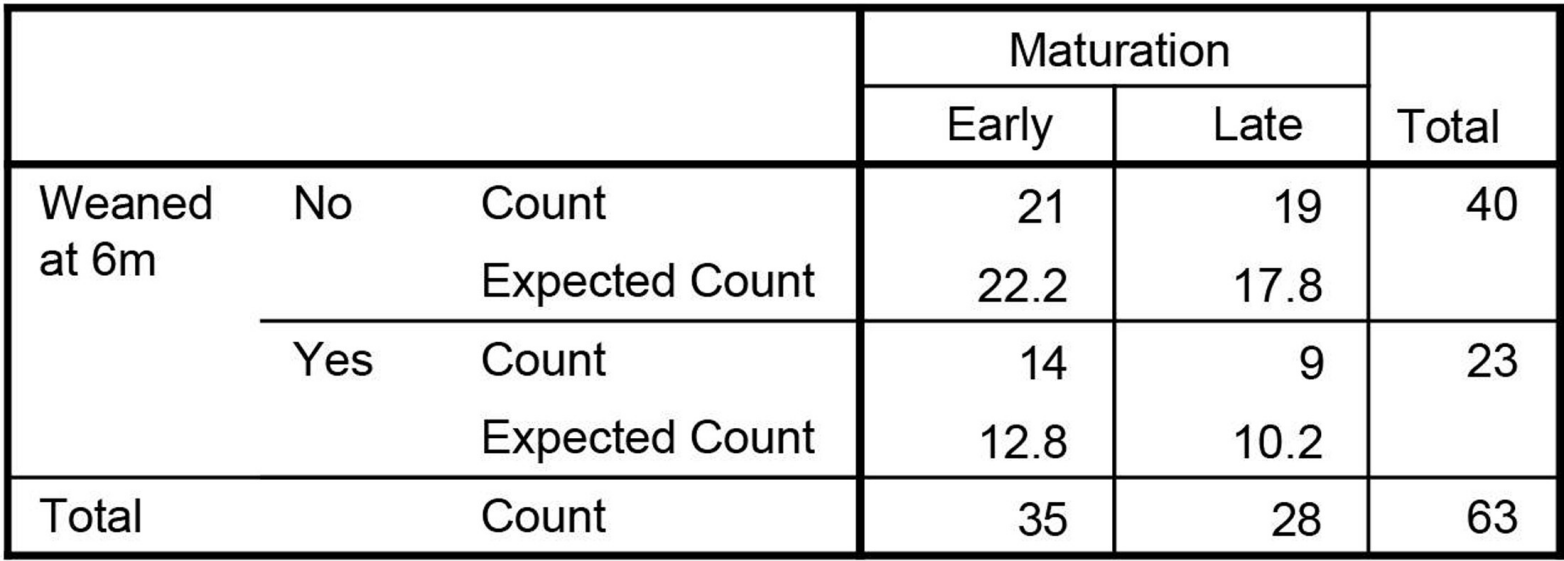
Maturation rate compared to expected values. The differences in maturation rate between timing of weaning and the expected values if it was random.

**Fig 12:**
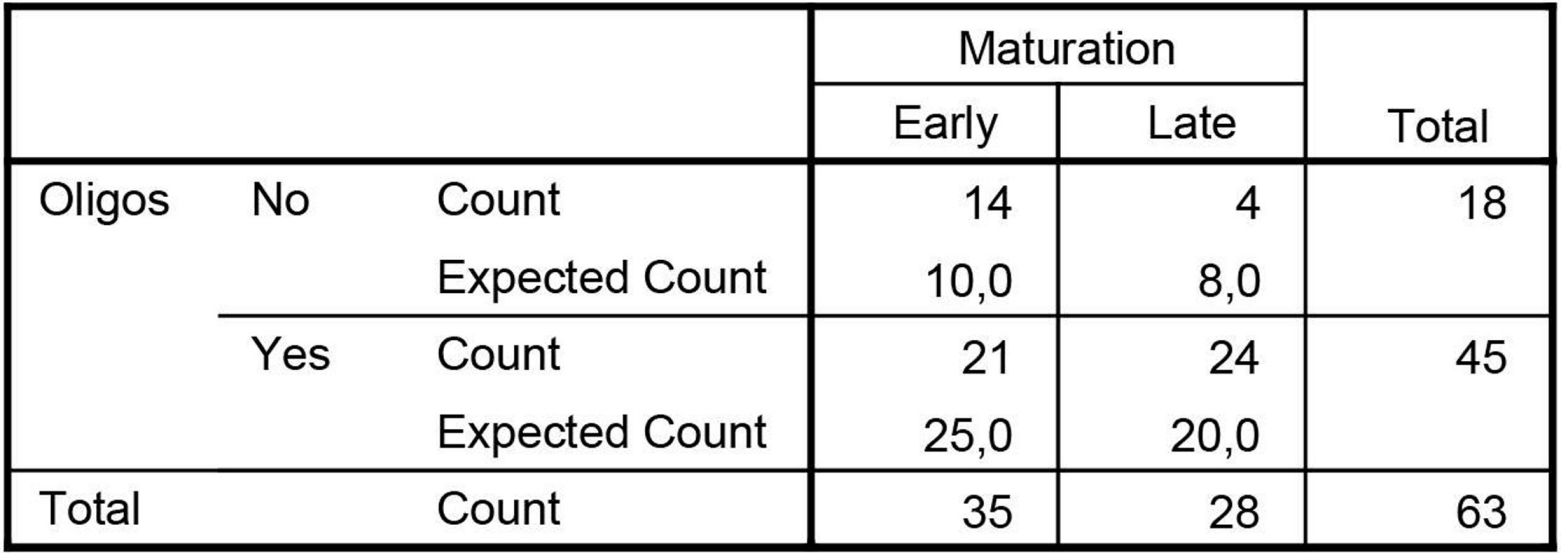
Maturation rate and diet oligos. The differences in maturation rate with our without oligos in the diet and the expected values if it was random.

We studied whether mode of feeding had influence on microbiota maturation. We regarded having a cluster 3 microbiota composition at the age of 6m as ‘early maturation’ and having a cluster 1 or 2 microbiota composition as ‘late maturation’. Early maturation is characterized by decreasing numbers of *Bifidobacterium* spp. and *Veillonella* spp. from 1m to 6m and increasing numbers of *Blautia* and *Lachnispiraceae Incertae Sedis* spp. (Fig 9). The abundance dynamics are highly intra-individual but overall we found different dynamics towards maturation. At 6m 44.4% of the infants had a ‘late maturation’ microbiota type (Fig 10). In breastfed infants the percentage of ‘late maturation’ was 87.5%. In formula fed and mixed feeding infants we found that 70.0% and 70.6% (respectively) had ‘early maturation’ as opposed to 55.6% among all the infants (Pearson Chi-Square test, p<0.001). We did not observe a significantly different distribution of microbiota maturation between infants that had started weaning and those that did not (Fig 11, Pearson Chi-Square test, p = 0.520). To test whether the presence of oligosaccharides in the nutrition of infants has an effect of on microbiota maturation, we divided our subjects in two groups. First a group that received oligosaccharides, either through human milk or prebiotics supplemented formula, and second a group that received formula without prebiotics (Fig 12). We found that the group that did not receive oligosaccharides more often had a more mature microbiota composition (Pearson Chi-Square test, p = 0.025). Clearly, we observed that early maturation is more common amongst formula fed infants, but late maturation is more common amongst prebiotics supplemented formula fed infants.

## Discussion

This study describes the microbiota development from a group of 64 children over the first 6 years of life. With these data we independently reproduced known concepts of microbiota development and come up with new findings, using a European cohort. Our dataset revealed two distinct types of early life microbiota composition; a typical *Bifidobacterium-*dominated [4, 31] and a more recently discovered *Bacteroides-*dominated infant microbiota [13, 19, 22]. The *Bacteroides-*dominated microbiota has been described previously in relation to birth mode. Vaginally born infants have been reported to have higher levels of *Bacteroides* and C-section born infants have been described to be depleted of *Bacteroides* [16, 19]. In our dataset none of the C-section born infants had a *Bacteroides-*dominated microbiota and as such this study supports previous findings. However, this study is limited because it is not able to link maternal comorbidities to the occurrence of C-section birth [32, 33]. Previous studies have actually shown that when adjusting for maternal comorbidities that include obesity and diabetes, birth mode delivers no discernable differences in infant gut microbiota composition [14].

All the infants in our study were receiving either human or formula milk feeding at 1m and 6m. It is at these time-points that the large majority of infants have a *Bifidobacterium-* or *Bacteroides-*dominated microbiota. Species from both genera are known to be well-equipped to metabolize the complex carbohydrates in milk [22, 34]. Because of their shared preference for these carbohydrates, *Bacteroides* spp. and *Bifidobacterium* spp. compete for dominance during the period that infants receive human milk. However, since we only report up to the phylogenetic family and genus level, the potential for human milk substrate utilization can still differ greatly between infants. For example, the *Bifidobacterium* spp. possess varied HMO utilization pathways [35]. Moreover, the relative abundance of the aforementioned genera can vary quite a lot between individuals. As such, the OTUs associated with either *Bacteroides* or *Bifidobacterium* in our study show differences between the early life microbiota clusters.

It has been previously described that the gut microbiota of infants can be dominated by *Bacteroides* spp. [23]. It appears that the dynamics of *Bacteroides* in the microbiota of some Belgian infants resembles that pattern. The *Bacteroides* spp. we find in cluster 2 are almost all related to *Bacteroides dorei* and *Bacteroides vulgatus*. When exploring associations of infant gut microbiota and food sensitization, a *Enterobacteriaceae*/*Bacteroidaceae* ratio was used as a measure for microbiota maturity [36]. Similarly, to this dataset, if the microbiota of infants is dominated by *Bacteroides* spp. at early infancy, their abundance might not reflect microbiota maturity as well as in cohorts without *Bacteroides-*dominated infants. Notably, on another cohort of Belgian children, this ratio was hypothesized to be a natural regulator of the Th1/Th2 balance, with the risk of early colonization by Bacteroides leading to a Th2 dominance and higher risk of asthma development [37].

The clustering analysis performed on samples of different ages allowed us to determine different phases in microbiota development. Subsequently analyzing the trajectories that individuals follow through the clusters that represent a maturation state led to the discovery of four distinct trajectories. Due to the number of samples, we were not able to make correlations of metadata variables with specific trajectories. However, the pooling of trajectories into either early or late maturation led to the finding that formula feeding is associated with early maturation in this cohort, thereby confirming previous observations by *Bäckhed et al*. [15]. If there is a difference in maturation among infants fed formula with and without prebiotics supplementation is not clear from this study.

Most infants have already reached the relatively stable microbiota state associated with a milk diet at the 1m timepoint. In a Singaporean study that also used a clustering approach to determine stages in microbiota development, but included samples taken at day 3, there is a cluster that is dominated by the family *Enterobacteriaceae*, especially *Klebsiella* spp. [38]. The study also finds a cluster with high levels of *Firmicutes*, in particular *Streptococcus*. We find *Streptococcus* to be the second most abundant genus in the *Bifidobacterium-*dominated microbiota and it rather seems to cooccur with *Bifidobacterium* than to compete with it. From the age of 3 weeks, the majority of infants in the Singaporean study had a *Bifidobacterium-*dominated microbiota similar to ours, indicating that our first time-point is probably too late to detect a *Enterobacteriaceae-*dominated infant microbiota cluster.

A study by Subramanian *et al*. analyzes the contribution of specific taxa to the microbiota age of Bangladeshi infants [24]. By calculating the correlation with age and analyzing the X-axis position in ordination plots, we find similar microbial groups. We also find that *Faecalibacterium* spp., *Ruminococcus* spp., *Bifidobacterium* spp., *Dorea* spp., *Staphylococcus* spp. and *Streptococcus* spp. are strongly correlated with age. An important difference is that we also find correlations for *Veillonella* spp., which might be a geographical effect.

Similarly to Bäckhed *et al*., we find increasing alpha diversity over time, combined with reduced beta-diversity [15]. We do not find increased diversity in terms of richness and Simpson’s index from 1m to 6m, even though 23 out of 63 infants had already started weaning at 6m. We did find increased phylogenetic diversity from 1m to 6m, indicating that although richness and evenness were similar, the phylogenetic distance between OTUs within the 6m samples increased. This demonstrates the suitability of phylogenetic diversity as compared to more classical diversity indices for this type of analysis.

At 6m, we did not find a difference in maturation state between infants that had already been weaned and infants that had not. Weaning often leads to transformation of the microbiota composition, since the solid foods that are introduced resemble the adult diet more than either human milk or formula [7, 39]. However, we did find that infants receiving milk with oligosaccharides, either breastmilk with HMOs or formula with added prebiotics possessed a pre-mature microbiota composition as compared to infants receiving formula without prebiotics. It seems that in this study, discontinuation of human and formula milk feeding with its associated oligosaccharides is more important than the introduction of solid foods. An observation also similar to that of Bäckhed *et al*. [15]. In conclusion, this study describes two types of infant gut microbiota ecosystems. This study features both the traditional *Bifidobacterium-*dominated infant microbiota and the microbiota dominated by *Bacteroides*. In our dataset, there were no C-section delivered infants that had a *Bacteroides* microbiota. Differential microbiota maturation was influenced by feeding mode. It seems that any milk diet and their oligosaccharide prebiotics delay microbiota maturation. Limitations of the study include the relatively small cohort size and the fact that it replicated previous findings. Furthermore, we cannot account for maternal confounding factors that might explain microbiota differences we observe in this cohort. Finally, the sequencing and subsequent bioinformatics analysis associated with this cohort was performed long before publication and further development of associated methods has occurred in the meantime. The next step will be to study the health effects of both infant microbiota types; early and late microbiota maturation and whether the presence of specific bacteria promotes or reduces infant health status.
